# Prospective evaluation of dynamic changes in L3 skeletal muscle index and chemotherapy-induced peripheral neuropathy during albumin-bound paclitaxel chemotherapy

**DOI:** 10.3389/fonc.2026.1844651

**Published:** 2026-07-27

**Authors:** Meijin Yue, Shuqin Deng, Gaowa Daleng, Feng Chen, Jun Zhao, Ying Jiang, Jiaxuan Li, Jiayuan Guo, Yalong Han, Zhengyu Li, Gaowa Jin, Quanfu Li

**Affiliations:** 1 Department of Medical Oncology, Ordos Central Hospital, Ordos, Inner Mongolia Autonomous Region, China; 2 Baotou Medical College, Inner Mongolia University of Science and Technology, Baotou, Inner Mongolia Autonomous Region, China; 3 Department of Geriatrics and General Internal Medicine, Ejin Horo Banner People’s Hospital, Ordos, Inner Mongolia Autonomous Region, China

**Keywords:** albumin-bound paclitaxel, body composition, chemotherapy-induced peripheral neuropathy, L3 skeletal muscle index, overall survival, sarcopenia

## Abstract

**Objective:**

This prospective study aimed to assess the association between dynamic changes in the L3 skeletal muscle index (L3SMI) and chemotherapy-induced peripheral neuropathy (CIPN) in patients receiving albumin-bound paclitaxel (nab-PTX)-based chemotherapy, and examine the impact of sarcopenia and CIPN on survival outcomes.

**Methods:**

Patients with malignancies scheduled to receive nab-PTX-based chemotherapy were enrolled. Nutritional status, body composition assessed by bioelectrical impedance analysis, and L3SMI measured using computed tomography were assessed at baseline and after three chemotherapy cycles. CIPN was assessed using the Patient Neurotoxicity Questionnaire after the first and third cycles. Patients were stratified according to baseline sarcopenia status and CIPN severity (high-grade, CIPN-H ≥ grade C; low-grade, CIPN-L < grade C).

**Results:**

Among the 152 enrolled patients, 56 completed all assessments. After three chemotherapy cycles, L3SMI decreased significantly from 32.77 to 31.93 cm²/m² (*p* = 0.049). CIPN severity increased over the course of treatment. The incidence of CIPN-H increased significantly from 10.71% to 39.29% (*p* < 0.001). The incidence of CIPN-H increased from 10.71% to 39.29% after three chemotherapy cycles. This increase was observed in both patients with and without sarcopenia, whereas the between-group difference after three cycles was not statistically significant. Median overall survival was significantly shorter in patients with CIPN-H (13 months) than in those with CIPN-L (24 months).

**Conclusion:**

Progressive loss of skeletal muscle-related body composition parameters during nab-PTX-based chemotherapy was accompanied by increased CIPN severity. CIPN-H was associated with poorer OS, but this association should be interpreted cautiously because of potential confounding by disease severity and treatment intensity. These findings support the importance of assessing body composition and implementing interventions targeting sarcopenia to reduce the risk of CIPN.

## 1 Introduction

From the *China Cancer Epidemiology Report 2022*, there were 4.825 million new cancer cases and 2.574 million cancer-related deaths annually in China, accounting for 24.2% and 18.3% of the global totals, respectively (1). Individuals aged ≥ 60 years contribute to more than 60% of the national cancer burden in China, with incidence and mortality rates that are 8.5-fold and 14.0-fold higher, respectively, than those observed in individuals aged < 60 years (2). These data indicate a substantial overlap between cancer and advanced age as coexisting conditions. The concept of sarcopenia originates from geriatric medicine. The Asian Working Group for Sarcopenia defines sarcopenia as an age-related reduction in skeletal muscle mass (SMM) accompanied by decreased muscle strength and/or impaired physical performance (3). A random-effects meta-analysis published in 2023, which included 65,936 patients with cancer, defined pre-treatment sarcopenia based on computed tomography (CT)-assessed muscle loss and reported a prevalence of 38.0%. The analysis further demonstrated that patients with sarcopenia experienced higher rates of treatment-related toxicity and poorer survival outcomes (4).

Nanoparticle albumin-bound paclitaxel (nab-PTX) is a nanoformulation of paclitaxel (PTX) that has demonstrated improved response rates and prolonged progression-free survival in patients with advanced metastatic breast cancer and non-small cell lung cancer, as well as extended overall survival (OS) in patients with advanced pancreatic cancer. However, chemotherapy-induced peripheral neuropathy (CIPN) remains one of the most common and dose-limiting toxicities associated with this agent (5–7). CIPN is characterized by sensory, motor, and/or autonomic dysfunction of varying severity. Sensory symptoms are typically most pronounced in the distal extremities and present in a characteristic “glove-and-stocking” distribution. These include tingling, numbness, impaired vibration perception, altered tactile sensation, paresthesia, and sensory disturbances triggered by touch or temperature changes; in severe cases, complete loss of sensation may occur (8). Motor symptoms occur less frequently than sensory symptoms and generally present as distal muscle weakness, impaired motor function, and disturbances in gait and balance (9). Currently, although multiple therapeutic strategies for CIPN have been proposed, including ion channel-targeted therapies, anti-inflammatory agents, and antioxidants, few have demonstrated definitive efficacy in the prevention and/or treatment of CIPN (10). The identification of risk factors associated with CIPN is of substantial clinical relevance and may inform individualized dosing strategies based on third lumbar vertebra (L3) skeletal muscle index (L3SMI). For example, a study published in JCO in 2025 reported a model for calculating oxaliplatin dosage based on lean body mass (LBM) in patients with colorectal cancer receiving adjuvant FOLFOX chemotherapy. This approach enabled the administration of a higher relative dose intensity (RDI) while maintaining survival benefits and significantly reducing oxaliplatin-induced neurotoxicity (11).

Shlomit et al. reported that sarcopenia in patients with metastatic breast cancer receiving taxane-based chemotherapy was associated with dose-limiting toxicity (DLT). However, that study included patients treated with PTX, nab-PTX, and docetaxel, and the toxicity assessment encompassed a broad range of post-chemotherapy adverse events (12). Prior studies conducted by this group demonstrated that patients with cancer and sarcopenia receiving nab-PTX chemotherapy experienced higher incidences of CIPN and severe CIPN, and that a higher dose of nab-PTX per kilogram of LBM may contribute to this increased risk (13). Tumor-associated sarcopenia requires comprehensive and longitudinal management throughout the course of treatment. Prior studies have shown that in patients with gastrointestinal cancer receiving XELOX/SOX regimens, the L3SMI progressively decreased during chemotherapy, accompanied by an increase in both the incidence and severity of chemotherapy-induced neutropenia (14–17). The aim of this study was to: (1) establish a quantitative association between dynamic changes in L3SMI and CIPN during nab-PTX chemotherapy; (2) examine a risk stratification model for CIPN based on baseline fat-free mass index (FFMI); and (3) provide a foundation for future clinical trials assessing individualized dose adjustments based on LBM. The precise novel contribution of this study lies in prospectively confirming the relationship between sarcopenia and CIPN during nab-PTX-based chemotherapy, providing both internal and external validation for a muscle mass-based dosing threshold.

## 2 Materials and methods

### 2.1 Study participants

#### 2.1.1 Study design

From September 2021 to June 2025, patients with pathologically confirmed malignant tumors admitted to the Department of Oncology at Ordos Central Hospital were prospectively enrolled. Chemotherapy with nanoparticle albumin-bound PTX (nab-PTX; 100 mg/vial, manufactured by Jiangsu Hengrui Pharmaceuticals Co., Ltd.) was administered as monotherapy or along with cisplatin (or carboplatin). Nab-PTX was administered according to two regimens: 260 mg/m² on day 1, or 130 mg/m² on days 1 and 8. Cisplatin was administered at 75 mg/m² divided over days 1–3, or carboplatin was administered on day 1 with a target area under the curve (AUC) of 5. Each treatment cycle lasted for 21 days.

Measurements were obtained at baseline and after three cycles of chemotherapy, including L3SMI assessed by CT, Nutritional Risk Screening 2002 (NRS2002), Patient-Generated Subjective Global Assessment (PG-SGA), and body composition assessed using bioelectrical impedance analysis (BIA). Patients were categorized into two groups: the baseline chemotherapy group (Group G1) and the post-chemotherapy group (Group G2). Concurrently, the incidence and severity of CIPN were assessed using the Chinese version of the Patient Neurotoxicity Questionnaire (PNQ) after one and three cycles of chemotherapy. Dynamic changes were assessed using a before-and-after self-controlled approach. The primary objective was to investigate the association between dynamic changes in L3SMI and the incidence and severity of CIPN during nab-PTX monotherapy or combination chemotherapy. Differences in survival outcomes among patients with varying CIPN severity were examined. This study was approved by the Medical Ethics Committee of Ordos Central Hospital (Approval No. 2024-130). This study was conducted in accordance with the declaration of Helsinki. Written informed consent was obtained from all participants. The study was registered with the Chinese Clinical Trial Registry (ChiCTR2500095637).

#### 2.1.2 Inclusion criteria

**Enrollment criteria:**

① Age ≥ 18 years, with a pathologically confirmed diagnosis of a malignant tumor, and scheduled to receive albumin-bound PTX monotherapy or combination chemotherapy with cisplatin (or carboplatin); ② Normal liver and kidney function and a normal electrocardiogram prior to chemotherapy, with biochemical parameters within the following ranges: white blood cell count > 3.5×10^9^/L, neutrophils > 1.5×10^9^/L, platelets > 85×10^9^/L, alkaline phosphatase ≤ 2.5 times the upper limit of normal (ULN), serum alanine aminotransferase and aspartate aminotransferase ≤ 3 times ULN, bilirubin ≤ 1.5 times ULN, and serum creatinine ≤ 1.5 times ULN; ③ Karnofsky Performance Status score ≥ 70; ④ Absence of hematological diseases; ⑤ No contraindications to the planned chemotherapy regimen; ⑥ No surgery or other interventions that could alter body composition during the period from the CT examination to the initiation of chemotherapy; ⑦ Ability to undergo regular follow-up assessments for peripheral neuropathy; ⑧ Receipt of adjuvant or first-line chemotherapy. For those receiving second-line chemotherapy, the subject must have no prior treatment with PTX or oxaliplatin, and the interval since the last chemotherapy must not exceed 12 months; ⑨ Ability to undergo regular follow-up assessments with BIA and CT; ⑩ Provision of written informed consent with a signed consent form.

#### 2.1.3 Exclusion criteria

① Known allergy to albumin-bound PTX or platinum-based drugs; ② Bedridden status at enrollment, with inability to cooperate with body composition measurements; ③ Presence of severe wasting diseases (such as tuberculosis, hyperthyroidism, or systemic lupus erythematosus); ④ Receipt of long-term hormone therapy (> 1 month); ⑤ Presence of conditions affecting drug excretion or metabolism, such as AIDS, kidney disease, or active hepatitis; ⑥ Discontinuation of anti-tumor treatment or failure to undergo regular follow-up assessments for peripheral neuropathy; ⑦ Individuals with bilirubin levels exceeding 1.5 times the upper limit of normal or serum creatinine levels exceeding 1.5 times the upper limit of normal; ⑧ Presence of liver, biliary, or pancreatic metastases that could potentially affect the metabolism of albumin-bound PTX; ⑨ Presence of diabetes mellitus or a history of peripheral neuropathy caused by prior chemotherapy or other factors (such as diabetic peripheral neuropathy, vitamin B deficiency, peripheral nerve injury, or carpal tunnel syndrome). These specific exclusions were implemented to minimize confounding factors and isolate the specific relationship between sarcopenia and nab-PTX-induced CIPN, ensuring a cleaner analysis of the primary variables.

### 2.2 Data collection

#### 2.2.1 Collection of basic information data

Basic patient data were collected at the time of first admission, including name, sex, age, place of origin, contact information, disease type, pathological stage, chemotherapy regimen, specific medication administration times, and other relevant clinical data.

#### 2.2.2 Measurement of height and weight

Height and weight were measured using standardized procedures prior to each chemotherapy cycle, and body mass index (BMI) and body surface area (BSA) were calculated using specified formulas.

The standardized procedure for height and weight measurement was as follows: ① The patient was instructed to stand upright on the measuring platform, with the chest extended and abdomen relaxed; ② Both heels were positioned together, with toes angled outward at approximately 60°, and the knees were maintained in a straight and aligned position; ③ The patient was instructed to look straight ahead, ensuring that the lower margin of the eye orbit and the upper margin of the external auditory canal were aligned in the same horizontal plane. The shoulders, buttocks, and heels simultaneously were maintained in contact with the support column, with the arms relaxed at the sides and palms facing the thighs; ④ The measurer’s line of sight was maintained at the level of the horizontal measuring plane during measurement recording. At each hospital admission, height and weight were measured according to standardized procedures, and BSA and BMI were calculated accordingly. BSA (m²) = [height (cm) + weight (kg) − 60]/100; BMI = weight (kg)/height² (m²).

#### 2.2.3 Nutritional scale assessment

Dynamic assessments using nutritional scales, BIA, and L3SMI were performed at baseline and after three cycles of chemotherapy, and the corresponding data were recorded:

Nutritional Risk Screening 2002 (NRS2002) and Patient-Generated Subjective Global Assessment (PG-SGA) assessment: All patients underwent scale-based assessments conducted by trained nutritionists within 48 hours of admission. The NRS2002 score was derived directly from the assessment tool (17). An NRS2002 score ≥ 3 was considered indicative of nutritional risk, warranting nutritional intervention, whereas a score < 3 was considered indicative of no nutritional risk; however, weekly screening during hospitalization was still required.

The PG-SGA assessment included both qualitative and quantitative components, with scores derived directly from the assessment tool (17). The classification criteria were as follows: A/≤ 1 point (well-nourished); B/2–8 points (suspected or moderately malnourished); C/≥ 9 points (severely malnourished).

L3SMI measurement: All patients underwent CT of the lower lumbar spine at the level of the L3 prior to chemotherapy. Muscle tissue was defined using a standard Hounsfield unit range of −29 to +150. Abdominal CT scans were performed using standard clinical non-contrast protocols with a slice thickness of 5 mm. For analysis, two consecutive cross-sectional images at the L3 vertebral level were selected to assess muscle area. Skeletal muscle area was calculated using a radiation therapy Treatment Planning System (XIO) software, including the psoas, quadratus lumborum, erector spinae, external oblique, internal oblique, and transversus abdominis muscles. The third lumbar vertebra is recognized as a key anatomical landmark for body composition assessment, as the proportions of skeletal muscle and adipose tissue at this level correlate with whole-body tissue composition. By analyzing CT images at the L3 level using imaging software and applying regression formulas, whole-body skeletal muscle mass, LBM, and the mass and distribution of various adipose tissue compartments can be estimated (18). Measurements derived from the L3SMI were considered applicable for the assessment of LBM. The formula for calculating the L3 skeletal muscle index was:

L3SMI= L3 vertebral skeletal muscle area (cm²)/height² (m²). The formula for calculating LBM was: LBM (kg) = 0.3 × [L3 vertebral skeletal muscle area (cm²)] + 6.06 (18).

Calculation of nab-PTX dose per kg LBM:The dose of nab-PTX per kg LBM was calculated using baseline CT-derived LBM. LBM was estimated according to the formula: LBM (kg) = 0.3 × L3 skeletal muscle area (cm²) + 6.06. For each patient, the planned total dose of nab-PTX per cycle was calculated according to the prescribed regimen and body surface area. For patients receiving nab-PTX 260 mg/m² on day 1, the planned per-cycle dose was calculated as 260 × BSA. For patients receiving nab-PTX 130 mg/m² on days 1 and 8, the planned per-cycle dose was also calculated as 260 × BSA. The dose per kg LBM was then calculated as follows:

nab-PTX dose per kg LBM (mg/kg LBM) = planned total nab-PTX dose per cycle (mg)/baseline CT-derived LBM (kg).

Baseline LBM was used because the purpose of this analysis was to evaluate pre-treatment body composition as a determinant of relative drug exposure. Data on actual cumulative dose, relative dose intensity, dose reductions, treatment delays, and reasons for dose modification were not systematically available; therefore, dose per kg LBM was calculated based on planned per-cycle dose rather than actual delivered dose intensity.

BIA measurement: Body composition measurements, including body fat mass, skeletal muscle mass, and body fat percentage, were performed in all enrolled patients using a multi-frequency bioelectrical impedance analyzer (Model: DBA-550) located in the Oncology Department of Ordos Central Hospital. Measurements were conducted under controlled ambient temperature conditions (20 °C–25 °C). Patients were required to fast and to empty the bladder and bowels prior to measurement. Measurements were avoided following exercise, after bathing, or during menstruation (19).

Follow-up for neurotoxicity: The incidence and severity of CIPN were assessed using the Chinese version of the PNQ (PNQ, Appendix 1). The assessment time window for neurotoxicity was defined as days 19 to 21 of the first chemotherapy cycle and days 19 to 21 following the third cycle of chemotherapy. Neurotoxicity was graded as follows: none (A); mild, not interfering with daily activities (B); moderate, not interfering with daily activities (C); moderate to severe, interfering with daily activities (D); and severe, accompanied by numbness and interfering with most daily activities (E). Based on definitions from prior studies, chemotherapy-induced neurotoxicity of grade C or higher was classified as the CIPN-H group, whereas neurotoxicity below grade C was classified as the CIPN-L group (20). To minimize reporting bias, all assessments were conducted by trained personnel following standardized procedures within predefined time windows. Assessors were not involved in CT-L3SMI and dose load calculations, thereby reducing information bias, although no formal blinding procedure was implemented.

### 2.3 Data analysis

Statistical analysis was performed using SPSS version 25.0. Measurement data conforming to a normal distribution were presented as mean ± standard deviation (x¯ ± s), and count data were presented as frequencies and percentages (n (%)). The main statistical methods were as follows:

Comparison between groups: Independent samples *t*-tests or paired *t*-tests (for before-and-after comparisons) were applied for continuous variables. The chi-square test or Fisher’s exact test was used for categorical variables, and the McNemar test was applied for paired categorical data.

Association and predictive analysis: Binary logistic regression was performed to assess factors associated with the occurrence of CIPN, with calculation of odds ratios (OR) and 95% confidence intervals (CI). For continuous predictor variables (e.g., FFMI), trend analysis was conducted after categorization into tertiles to assess dose–response relationships. The discriminatory performance of predictive indicators was assessed using receiver operating characteristic (ROC) curve analysis, and the AUC was calculated. Based on the logistic regression results, absolute risk reduction (ARR) and number needed to treat (NNT) were calculated for the high-risk group compared with the low-risk group. Survival analysis: Survival curves were generated using the Kaplan–Meier method, and comparisons between groups were performed using the log-rank test. Univariate and multivariate Cox proportional hazards models were applied to identify independent prognostic factors, with calculation of hazard ratios (HR) and 95% CI. Missing data were handled using a complete-case approach. The final longitudinal analysis included only patients with complete paired assessments at baseline and after three cycles of chemotherapy, including CT-derived L3SMI, BIA-derived body composition, nutritional assessments, and PNQ-based CIPN evaluation. Therefore, no imputation was performed for longitudinal endpoints. For survival analyses, patients without available OS time were excluded from Kaplan–Meier and Cox regression analyses. Sensitivity analyses were performed using non-parametric paired tests to examine whether the direction and significance of longitudinal changes were consistent with the primary analyses.

All statistical tests were two-sided, and *p* < 0.05 was considered statistically significant.

## 3 Results

### 3.1 Baseline characteristics of the enrolled participants

From September 2021 to June 2025, a total of 152 patients were enrolled in the study, of whom 56 completed dynamic follow-up assessments, Among the 56 patients included in the final longitudinal cohort, no missing data were observed for the core paired variables, including CT-derived L3SMI, BIA-derived body composition, nutritional assessments, and PNQ-based CIPN evaluation. Therefore, all 56 patients were included in the longitudinal analyses, including nutritional scales, L3SMI, BIA, and CIPN assessments. This cohort included 43 males and 13 females. As presented in **Table 1**, 76.8% (43/56) of the patients were male, with a mean age of 65.39 ± 8.20 years and a mean follow-up duration of 117.62 ± 43.38 days.

**Table 1:** Clinical characteristics of patients.

Characteristics	A (N = 32)	B (N = 24)	P_value
Age (years)	65.2 ± 8.2	65.6 ± 8.3	0.852
Gender (Male/Female)	25 vs. 7	18 vs. 6	0.768
Height (cm)	168.9 ± 8.1	168.1 ± 6.0	0.692
Weight baseline (kg)	60.2 ± 9.2	69.2 ± 10.1	**0.001**
NRS2002 score	2.9 ± 1.9	1.9 ± 1.3	0.018
PG-SGA Scale	9.59 ± 4.78	4.92 ± 4.39	**<0.001**
≤1 score [n(%)]	1 (3.12%)	0 (0%)	
2–8 scores [n(%)]	13 (40.62%)	11 (45.83%)	
≥9 scores [n(%)]	18 (56.25%)	13 (54.17%)	
L3SMA (cm²)	79.0 ± 15.5	110.3± 27.6	**<0.001**
L3SMI (cm²/m²)	27.6 ± 4.7	38.7 ± 8.5	**<0.001**
Body mass index(kg/m2)			<0.001
<18.5 Underweight	8 (25.0%)	0 (0.0%)	
18.5-23.9 Normal	21 (65.6%)	10 (41.7%)	
24.0-27.9 Overweight	2 (6.2%)	12 (50.0%)	
≥28.0 Obese	1 (3.1%)	2 (8.3%)	
LBM (kg)	29.7 ± 4.7	39.1 ± 8.3	**<0.001**
FFMI (kg/m²)	16.3 ± 1.9	18.0 ± 1.8	**<0.001**
Cancer type [n(%)]			0.605
Lung cancer	13 (40.6%)	13 (54.2%)	
Gastrointestinal cancer	15 (46.9%)	8 (33.3%)	
Others	4 (12.5%)	3 (12.5%)	
Treatment regimen [n(%)]			0.845
nab-PTX monotherapy	18 (56.2%)	15 (62.5%)	
nab-PTX combination with cisplatin/carboplatin	14 (43.8%)	9 (37.5%)	

Bold indicates P < 0.05.

The remaining 96 patients did not complete all assessments: 35 did not complete follow-up CT after three cycles, 12 did not complete BIA or nutritional reassessment, 8 discontinued treatment or changed regimen before three cycles, 9 experienced disease progression or died before reassessment, 14 were transferred to another hospital or lost to follow-up, and 18 had COVID-19-related interruption of scheduled follow-up or restricted hospital access. No patient was excluded because of incomplete PNQ assessment. The patient selection process is shown in **Supplementary Figure 1**. Available baseline characteristics of patients included and not included in the final longitudinal analysis are compared in **Supplementary Table 1**.

Based on the Asian consensus criteria for the evaluation of sarcopenia in patients with cancer (male L3SMI < 36 cm²/m²; female L3SMI < 29 cm²/m²), patients were classified into a sarcopenia group (Group A) and a non-sarcopenia group (Group B) (21). The incidence of sarcopenia was 57.14% (32/56). As presented in **Table 1**, no statistically significant differences were observed between the non-sarcopenia group (Group B) and the sarcopenia group (Group A) with respect to baseline characteristics, including age, sex, height, disease type, and chemotherapy regimen.

Patients in Group A demonstrated significantly lower values for weight, NRS2002 score, PG-SGA score, BMI, L3SMI, LBM, and FFMI (*p* < 0.05). BMI classification was performed in accordance with the *Guidelines for Nutritional Treatment of Cancer in China 2020* (17): (1) BMI <18.5 kg/m², underweight (malnourished); (2) BMI 18.5–23.9 kg/m², normal weight; and (3) BMI 24–27.9 kg/m², overweight. Notably, all underweight patients (BMI < 18.5 kg/m²) were observed in Group A (25.0%, 8/32), whereas patients in Group B were predominantly overweight or obese (58.3%, 14/24).

### 3.2 Dynamic changes in nutritional status indicators prior to and following chemotherapy

**Figure 1:**
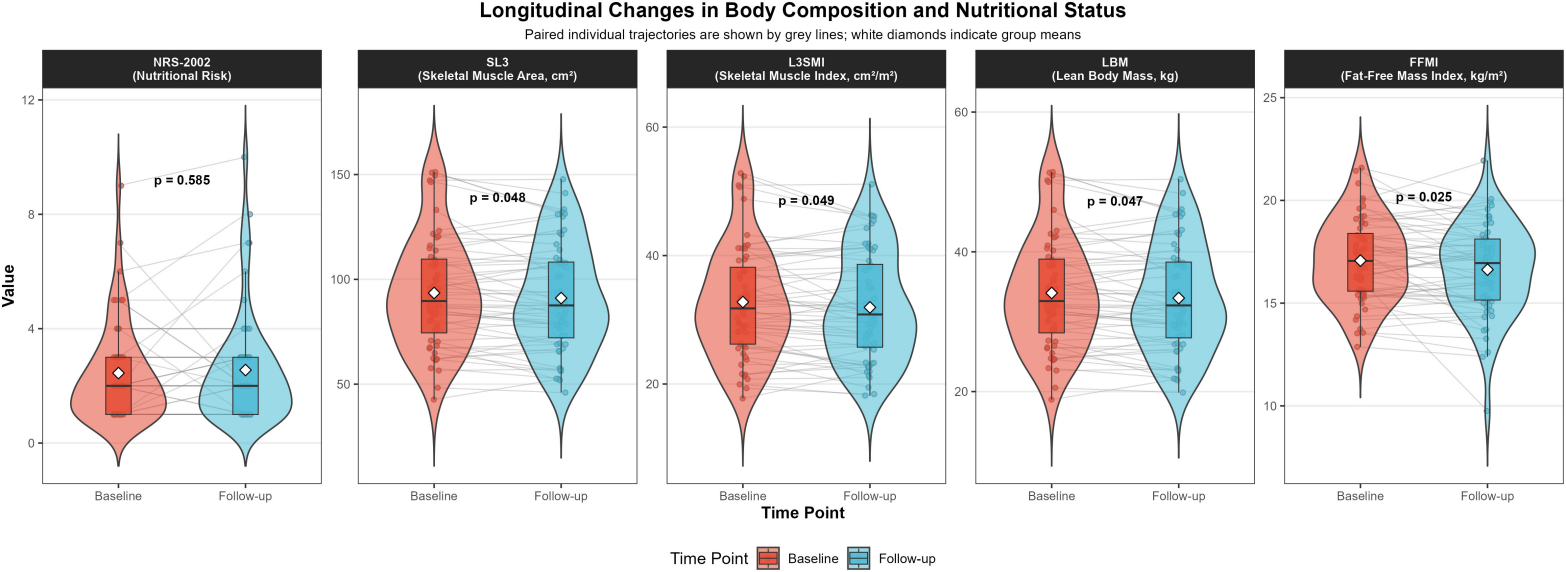
Trends changes of nutritional status-related indicators.

Baseline nutritional assessment prior to chemotherapy was defined as Group G1, and nutritional status assessed after three cycles of chemotherapy was defined as Group G2. As depicted in **Figure 1** (violin plot), NRS2002 scores presented a slight increase from baseline to after three cycles of chemotherapy. In contrast, CT-derived skeletal muscle area at L3 (L3SMA), L3SMI, and body composition parameters, including LBM and FFMI, demonstrated a progressive and statistically significant decline over the course of chemotherapy. As presented in **Table 2**, no statistically significant changes were observed in nutritional scale indicators after three cycles of chemotherapy, including NRS2002 (*p* = 0.585), PG-SGA (*p* = 0.250), and BMI (*p* = 0.934). However, body composition parameters measured by CT and BIA showed significant reductions.

**Table 2:** Comparison of nutrition status between G1 and G2.

	G1	G2	Percent Change (%)	*P*-value	*(95% CI)*
NRS 2002	2.45 ± 1.70	2.55 ± 1.93	+4.08 ± 35.92	0.585	0.11 (−0.28 to 0.50)
PG-SGA	7.59 ± 5.14	8.27 ± 5.41	+8.96 ± 28.76	0.250	0.68 (−0.49 to 1.85)
L3SMA(cm²)	93.51 ± 26.89	91.06 ± 25.83	-2.62 ± 4.47	**0.048**	0.02 (−0.48 to 0.52)
L3SMI (cm²/m²)	32.77 ± 8.72	31.93 ± 8.46	-2.56 ± 4.89	**0.049**	−2.45 (−4.86 to −0.03)
Weight (kg)	64.41 ± 10.57	64.25 ± 11.34		0.848	−0.84 (−1.67 to −0.00)
BMI (kg/m²)	22.68 ± 3.43	22.70 ± 3.51	+0.09 ± 1.54	0.934	−0.73 (−1.46 to −0.01)
LBM (kg)	34.11 ± 8.07	33.38 ± 7.75	-2.14 ± 4.62	**0.047**	−0.44 (−0.83 to −0.06)
FFMI (kg/m²)	17.07 ± 2.04	16.63 ± 2.23	-6.09 ± 6.18	**0.025**	−0.00 (−0.01 to 0.01)

Data are mean ± SD. L3SMI, L3 skeletal muscle index; FFMI, fat-free mass index; L3SMA, skeletal muscle area at L3; LBM, lean body mass. Bold indicates P < 0.05 *.

As presented in **Table 2**, no statistically significant changes were observed in nutritional scale indicators after three cycles of chemotherapy, including NRS2002 (mean paired difference: 0.11, 95% CI: −0.28 to 0.50, p = 0.585), PG-SGA (mean paired difference: 0.68, 95% CI: −0.49 to 1.85, p = 0.250), and BMI (mean paired difference: 0.02 kg/m², 95% CI: −0.48 to 0.52, p = 0.934).

In the primary paired t-test analyses, body composition parameters measured by CT and BIA showed modest reductions. Specifically, L3SMA decreased from 93.51 ± 26.89 cm² to 91.06 ± 25.83 cm², with a mean paired difference of −2.45 cm² (95% CI: −4.86 to −0.03, p = 0.048). L3SMI decreased from 32.77 ± 8.72 cm²/m² to 31.93 ± 8.46 cm²/m², with a mean paired difference of −0.84 cm²/m² (95% CI: −1.67 to −0.00, p = 0.049). LBM decreased from 34.11 ± 8.07 kg to 33.38 ± 7.75 kg, with a mean paired difference of −0.73 kg (95% CI: −1.46 to −0.01, p = 0.047). FFMI decreased from 17.07 ± 2.04 kg/m² to 16.63 ± 2.23 kg/m², with a mean paired difference of −0.44 kg/m² (95% CI: −0.83 to −0.06, p = 0.025).

### 3.3 Sarcopenia group vs. non-sarcopenia group

In the overall study population, the dynamic change in each body composition parameter was defined as Δ. As presented in **Table 3**, no statistically significant differences were observed in the magnitude of dynamic changes between the sarcopenia group (Group A) and the non-sarcopenia group (Group B) (*p* > 0.05). However, greater absolute decreases in L3SMI, LBM, and FFMI were observed in Group B compared with Group A.

**Table 3:** Comparison of nutrition status dynamics changes between A and B.

	AΔ	BΔ	*P*_value
**ΔPG-SGA (score)**	1.1 ± 3.69	0.19 ± 5.07	0.345
**ΔNRS2002 (score)**	0.1 ± 1.40	0.12 ± 1.56	0.407
**ΔL3SMA(cm²)**	-1.73 ± 7.40	-3.27 ± 10.7	0.889
**ΔL3SMI (cm²/m²)**	-0.65 ± 2.65	-1.06 ± 3.63	0.626
**ΔWeight (kg)**	-0.47 ± 5.08	0.82 ± 4.80	0.895
**ΔBMI (kg/m²)**	-0.21 ± 1.99	0.29 ± 1.70	0.902
**ΔLBM (kg)**	-0.52 ± 2.22	-0.98 ± 3.21	0.870
**ΔFFMI (kg/m²)**	-0.25 ± 1.25	-0.66 ± 1.61	0.200

Δ: change in BIA from baseline to cycle 3. Bold indicates P < 0.05.

### 3.4 Dynamic changes in CIPN during chemotherapy

**Figure 2:**
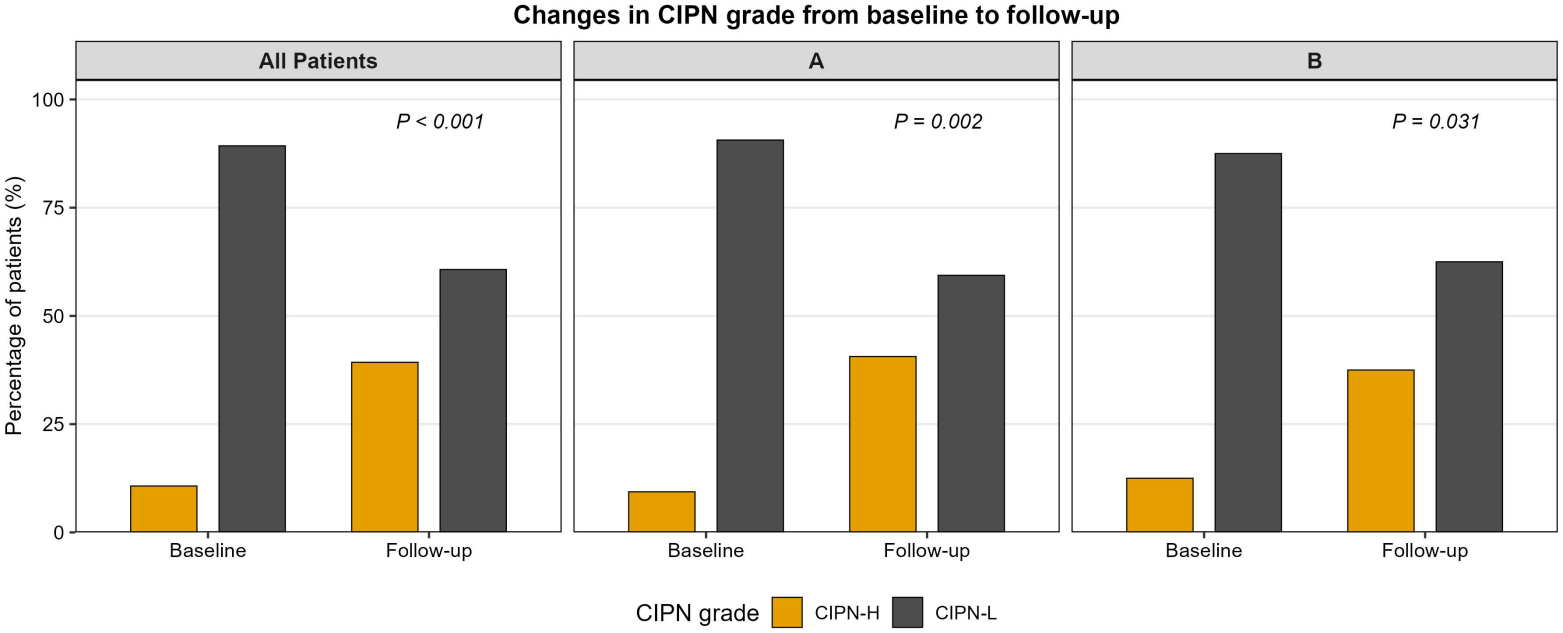
CIPN incidence dynamic changes during chemotherapy.

Among the 56 patients who completed dynamic assessments, the severity of CIPN increased significantly with successive treatment cycles. The incidence of CIPN-H (≥ grade C) increased significantly from 10.71% (6/56) at baseline to 39.29% (22/56) (*p* < 0.001, 95% CI: 17.86% to 41.07%), whereas the incidence of CIPN-L (< grade C) decreased from 89.29% (50/56) to 60.71% (34/56) (*p* < 0.001). As presented in **Figure 2**, dynamic changes in CIPN were compared between the sarcopenia group (Group A) and the non-sarcopenia group (Group B). After three cycles of chemotherapy, the incidence of CIPN-H increased significantly in both groups: Group A [9.38% (3/32) vs. 40.63% (13/32), *p* = 0.002] and Group B [12.50% (3/24) vs. 37.50% (9/24), *p* = 0.031]. The incidence of CIPN-L in Group A decreased from 90.63% (29/32) to 59.37% (19/32), which was statistically significant (*p* = 0.002). Similarly, in Group B, CIPN-L decreased from 87.50% (21/24) at baseline to 62.50% (15/24) (*p* = 0.031).

After three cycles of chemotherapy, no statistically significant difference in the incidence of CIPN-H was observed between the two groups (40.63% vs. 37.50%, *p* = 0.816). The magnitude of increase from baseline to three cycles was greater in the sarcopenia group (Group A) (+31.3%) compared with the non-sarcopenia group (Group B) (+25.0%).

### 3.5 Dose intensity of nab-paclitaxel per unit of lean soft tissue

**Figure 3:**
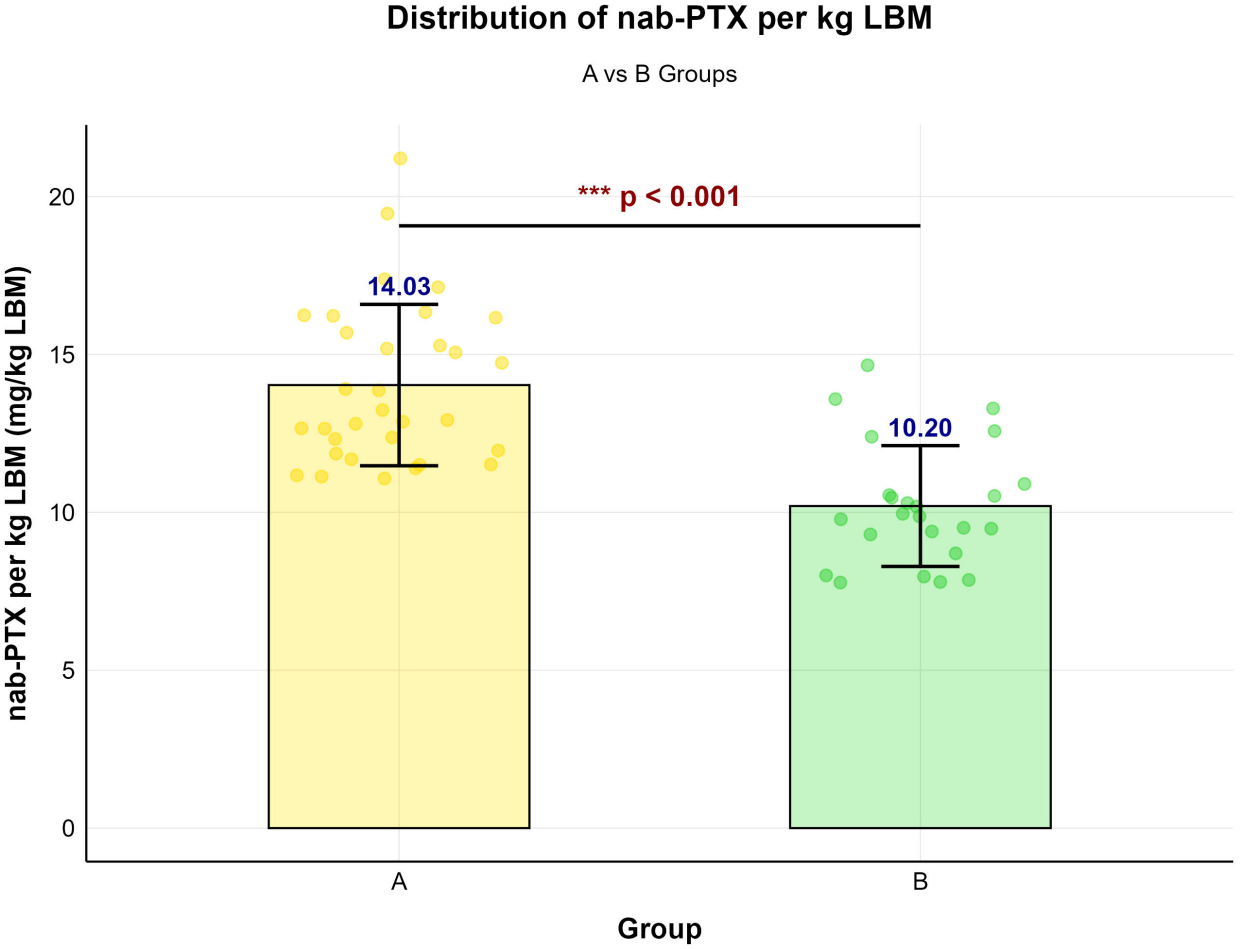
Distribution of Nab-PTX mg/kg LBM between A and B.

From the formula for calculating LBM: LBM (kg) = 0.3 × [L3 vertebral skeletal muscle area (cm²)] + 6.06, patients in the sarcopenia group (Group A) and the non-sarcopenia group (Group B) were standardized according to LBM (18). Comparison of the nab- PTX (nab-PTX) dose per kilogram of LBM (mg/kg LBM) between Group A and Group B demonstrated that Group A received a significantly higher mean nab-PTX dose per kg LBM than Group B (14.03 mg/kg LBM vs. 10.20 mg/kg LBM, *p* < 0.001) (**Figure 3**).

**Figure 4:**
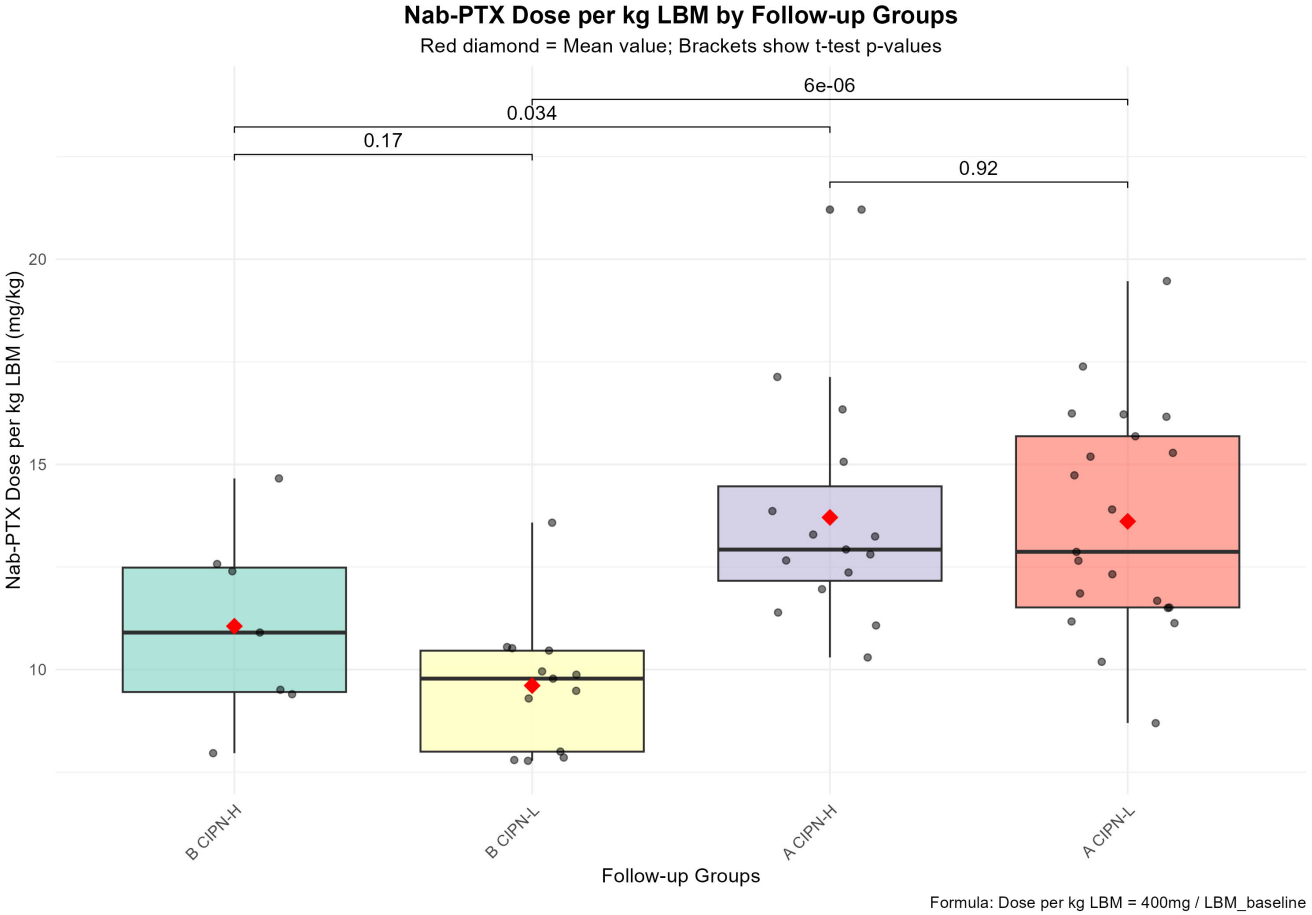
The relationship of Nab-PTX mg/kg LBM And CIPN.

As shown in **Figure 4**, among patients with CIPN-H after three cycles of chemotherapy, the dose intensity of nab-PTX (mg/kg LBM) in Group A was significantly higher than that in Group B (14.2 mg/kg LBM vs. 10.9 mg/kg LBM, *p* = 0.034). Similarly, among patients with CIPN-L, the dose intensity of nab-PTX (mg/kg LBM) in Group A was significantly higher than that in Group B (14.0 mg/kg LBM vs. 9.6 mg/kg LBM, *p* < 0.001). However, in within-group comparisons for Group A and Group B, although the dose intensity of nab-PTX (mg/kg LBM) was higher in the CIPN-H subgroup than in the CIPN-L subgroup, the differences did not reach statistical significance.

### 3.6 Survival analysis

For survival analyses, OS data were unavailable in 2 patients. These patients were not included in the Kaplan–Meier and Cox regression analyses because their OS time could not be reliably determined. In the multivariate Cox regression analysis (**Table 4**), variables with *p* < 0.1 in the univariate analysis (presented in **Supplementary Table 2**) were included after adjustment for other covariates, including CIPN-H, cancer type, and use of platinum-based combination chemotherapy. CIPN-H was remained associated with poorer OS after adjustment for available covariates, including cancer type and platinum-based combination chemotherapy. The risk of death in patients in the CIPN-H group was 3.462 times that of patients in the CIPN-L group (95% CI: 1.631–7.347, *p* = 0.001). When compared with patients with digestive system cancers, patients with other cancer types exhibited a significantly increased risk of death (HR = 4.691, 95% CI: 1.165–18.886, *p* = 0.030). In contrast, compared with patients receiving monotherapy, those receiving combination chemotherapy with platinum agents demonstrated a significantly reduced risk of death (HR = 0.357, 95% CI: 0.145–0.884, *p* = 0.026). No statistically significant difference in survival was observed between patients with lung cancer and those with digestive system cancers in the multivariate analysis (HR = 0.557, 95% CI: 0.243–1.278, *p* = 0.167).

**Table 4:** Multivariate Cox regression analysis for overall survival.

Variable	Hazard ratio (95% CI)	P-value
CIPN-H		
Yes vs. No	3.462 (1.631-7.347)	0.001
Cancer type		
Lung cancer vs. Digestive system cancer	0.557 (0.243-1.278)	0.167
Other vs. Digestive system cancer	4.691 (1.165-18.886)	0.030
nab-PTX in combination with cisplatin/carboplatin		
Yes vs. No	0.357 (0.145-0.884)	0.026

CI, confidence interval.

**Figure 5:**
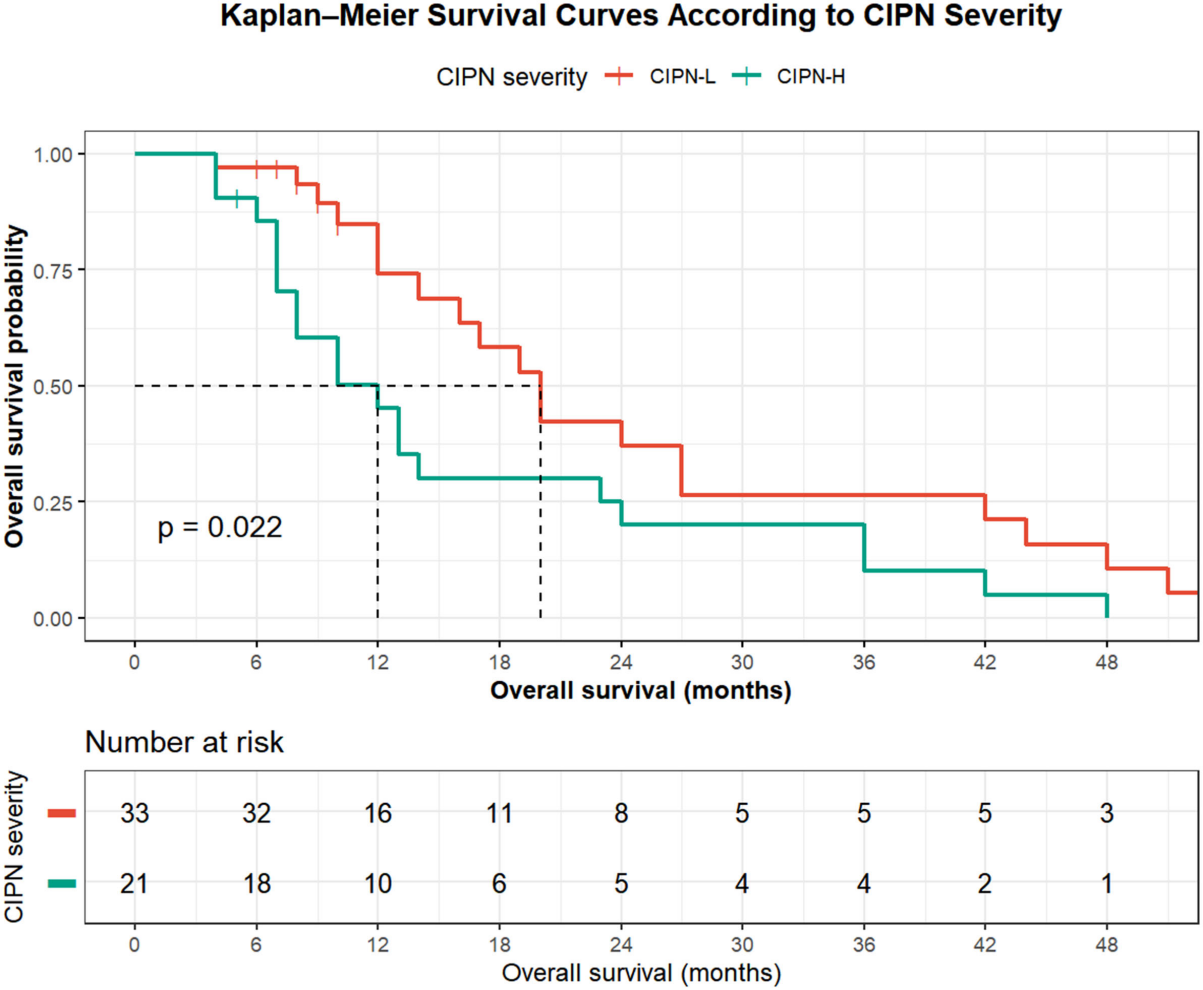
Kaplan-Meier survival curves by CIPN severity. Kaplan-Meier curves comparing overall survival between patients with high-grade (CIPN-H) and low-grade (CIPN-L) chemotherapy-induced peripheral neuropathy. Log-rank test P-value is shown. The shaded areas represent 95% confidence intervals.

The survival curves (**Figure 5**) demonstrated a significant impact of CIPN severity on prognosis. The median OS of patients in the CIPN-H group (13 months; 95% CI: 7.08–18.92) was significantly shorter than that of patients in the CIPN-L group (24 months; 95% CI: 14.47–33.53, *p* = 0.022).

### 3.7 Association between FFMI and CIPN

Multivariate logistic regression and trend analysis demonstrated that baseline FFMI was an independent protective factor against CIPN. Higher FFMI levels were significantly associated with a lower risk of severe CIPN and a reduced likelihood of CIPN grade progression. Analysis based on FFMI tertiles showed that the incidence of severe CIPN (CIPN-H) was 21.1% in the high FFMI group, which was significantly lower than the 57.9% observed in the low FFMI group (test for trend *p* = 0.024, OR = 0.442), indicating a dose–response relationship (AUC = 0.682, 95% CI: 0.536–0.829). The optimal cutoff value determined by the Youden index was 17.55 kg/m², with a sensitivity of 81.8% and specificity of 55.9% (Youden index = 0.377). These findings suggest that FFMI may serve as an exploratory risk stratification marker, although its discriminative performance was moderate and requires validation in larger cohorts.

**Figure 6:**
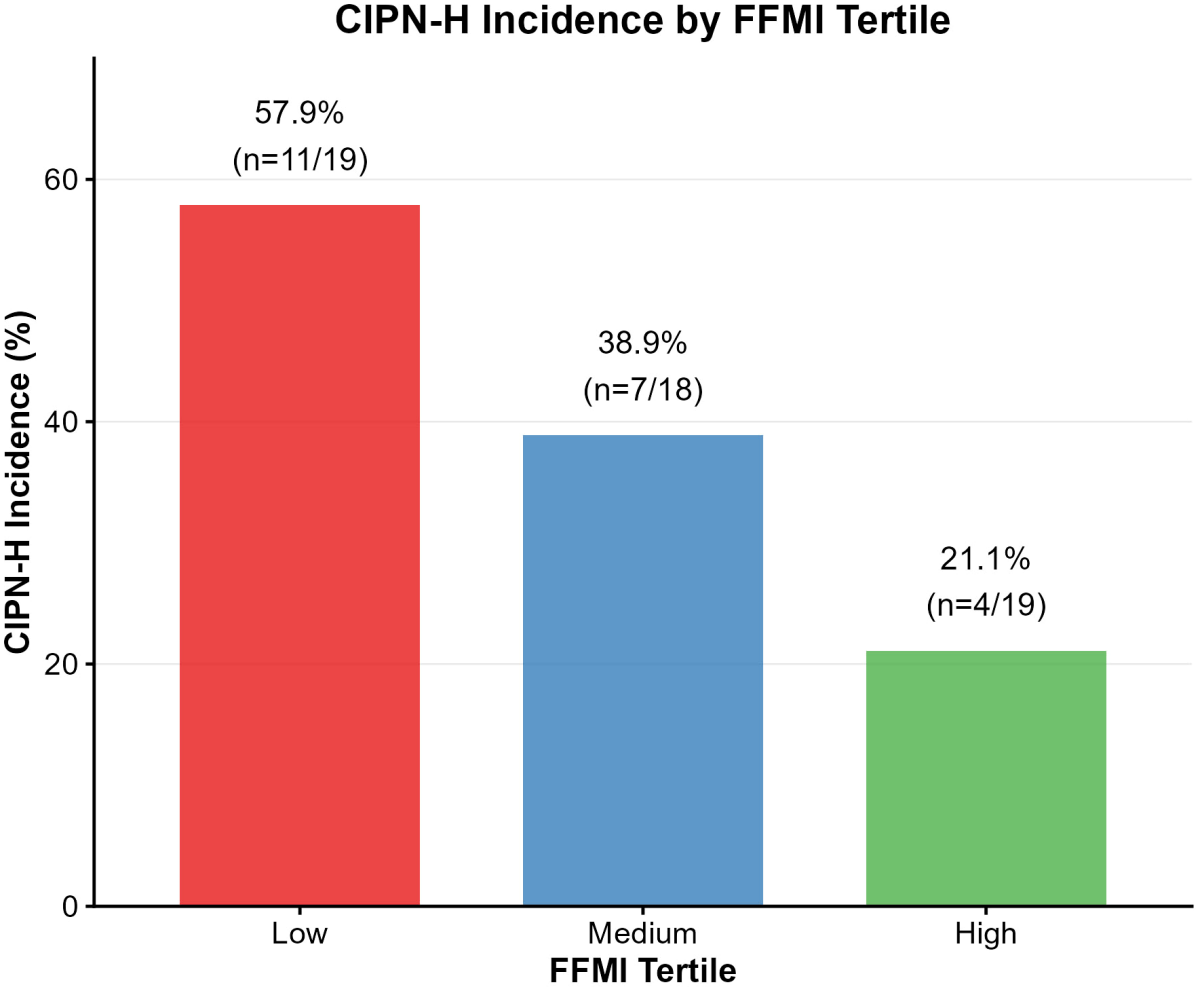
CIPN-H incidence by FFMI tertile.

Based on these findings, patients were stratified into three risk categories, as illustrated in **Figure 6**: low-risk (FFMI > 18.5), intermediate-risk (FFMI 16.0–18.5), and high-risk (FFMI < 16.0). Specifically, the incidence of CIPN-H in the low-, intermediate-, and high-risk groups was 57.9%, 38.9%, and 21.1%, respectively.

Compared with the low FFMI group, the high FFMI group demonstrated a 63.6% relative risk reduction for CIPN (ARR of 36.8%), corresponding to an NNT of 2.7.

## 4 Discussion

This prospective dynamic monitoring study assessed longitudinal changes in the L3SMI during nab-PTX chemotherapy and their association with CIPN. The main findings are summarized as follows: ① After three cycles of chemotherapy, L3SMI, LBM, and FFMI were significantly reduced; ② The incidence of severe CIPN (grade ≥ C) increased from 10.7% at baseline to 39.3%, with a greater increase observed in the sarcopenia group compared with the non-sarcopenia group; ③ With or without CIPN-H or CIPN-L classification, patients in the sarcopenia group received a relatively higher nab-PTX dose per kilogram of LBM (mg/kg LBM); ④ Severe CIPN was identified as an independent adverse predictor of OS (HR = 3.46); ⑤ Baseline FFMI demonstrated a significant inverse dose–response relationship with the risk of severe CIPN, and patients with high FFMI (> 18.5 kg/m²) exhibited a 63.6% reduction in the risk of CIPN-H. These findings indicate a concurrent progressive decline in L3SMI and an increase in the incidence of grade C CIPN during nab-PTX chemotherapy. Although international guidelines recommend assessing muscle function (e.g., grip strength or gait speed) for sarcopenia diagnosis, the CSCO guidelines recommend CT-derived L3SMI as the gold standard for diagnosing sarcopenia in oncology patients. Our research group has long focused on sarcopenia and previously found inconsistencies between different assessment methods; therefore, we directly utilized the CT-derived gold standard.

In this study, L3SMI decreased from 32.77 cm²/m² at baseline to 31.93 cm²/m² after three cycles of nab-PTX chemotherapy, representing a reduction of 2.56% (*p* = 0.049). These findings further support the observation that L3SMI progressively declines with successive chemotherapy cycles. Kurk et al. analyzed data from the phase III randomized controlled CAIRO3 study in advanced colon cancer and reported that SMM decreased during the initial six cycles of CAPOX-BEV (capecitabine, oxaliplatin, and bevacizumab) chemotherapy, increased during subsequent maintenance therapy or observation, and decreased again following disease progression when treatment was intensified (21). These results are consistent with the present findings.

Similarly, findings consistent with these observations have been reported in previous studies conducted in gastrointestinal tumors. Li et al. enrolled 39 patients with gastric cancer receiving XELOX/SOX chemotherapy and reported a decrease in L3SMI from 36.00 cm²/m² to 34.99 cm²/m² after three cycles, corresponding to a reduction of approximately 2.8% (*p* = 0.043) (16). Although differences existed between the two studies in terms of chemotherapy regimens (taxanes vs. platinum plus fluoropyrimidines) and tumor types (mixed tumors vs. gastric cancer), both demonstrated progressive reductions in L3SMI over successive chemotherapy cycles, with comparable rates of muscle loss after three cycles. These observations suggest that chemotherapy-associated sarcopenia may represent a treatment-related complication that is not limited to specific chemotherapeutic agents.

The magnitude of reduction observed in this study differs from that reported in some previous studies. Park et al. followed patients receiving palliative chemotherapy for advanced gastric cancer for up to six cycles and reported an L3SMI reduction of 11.3% (8). Yamaoka et al. followed patients receiving adjuvant chemotherapy after gastric cancer surgery for 12 months and reported a reduction of 6.2% (22). These differences may be attributable to variations in the duration of the observation period and the timing of baseline assessment. In the present study, follow-up was limited to three chemotherapy cycles, and muscle loss is known to exhibit a time-dependent cumulative effect.

In contrast, Yamaoka et al. used the postoperative period as baseline, during which surgical stress may induce acute muscle catabolism, with subsequent chemotherapy further exacerbating this effect. Notably, Suzuki et al. used a comparable observation window (2–3 cycles) in patients with unresectable pancreatic cancer receiving nab-PTX plus gemcitabine chemotherapy and reported decreases in SMI of 6.5% in the non-sarcopenia group and 4.4% in the sarcopenia group (*p* = 0.084) (23).

In this study, using a similar three-cycle observation period, a consistent pattern was observed, with a greater absolute reduction in muscle mass in the non-sarcopenia group compared with the sarcopenia group (−1.06 ± 3.63 vs. −0.65 ± 2.65). Despite differences in patient populations, tumor types, and chemotherapy regimens between the present study and that of Suzuki et al., both studies demonstrated that early chemotherapy-associated muscle loss may not be dependent on baseline muscle reserves, and that patients without sarcopenia may experience a greater magnitude of early muscle loss.

In this study, the non-sarcopenia group demonstrated better baseline PG-SGA scores than the sarcopenia group (PG-SGA 4.92 vs. 9.59, *p* < 0.001), and no significant increase in nutritional scores was observed during chemotherapy. This finding may be associated with structured nutritional management during hospitalization. A retrospective study conducted in 2024 in Korea reported that, in older patients with locally advanced esophageal cancer receiving neoadjuvant chemoradiotherapy, muscle loss was an independent risk factor for poorer OS, whereas baseline muscle mass was not significantly associated with survival outcomes (24). Taken together with the findings of this study, these results support the clinical importance of early and proactive implementation of nutritional and exercise interventions for sarcopenia.

The primary endpoint of this study was the association between dynamic changes in L3SMI during nab-PTX chemotherapy and the incidence and severity of CIPN. As L3SMI progressively declined during chemotherapy, a concurrent increase in both the incidence and severity of CIPN was observed. The incidence of severe CIPN (grade ≥ C) increased significantly from 10.71% (6/56) at baseline to 39.29% (22/56) after three cycles (*p* < 0.001), whereas the proportion of mild-to-moderate CIPN (< grade C) decreased from 89.29% to 60.71% (*p* < 0.001). The increase in CIPN-H was more pronounced in the sarcopenia group, with an absolute increase of 31.26% (9.38% vs. 40.63%, *p* = 0.002), compared with 25.00% (12.5% vs. 37.5%, *p* = 0.031) in the non-sarcopenia group, suggesting greater susceptibility to the cumulative neurotoxic effects of taxane-based chemotherapy among patients with sarcopenia.

Prior studies have demonstrated an association between chemotherapy dose intensity per unit of LBM and treatment-related toxicities. Ali et al. reported that among patients with colon cancer receiving FOLFOX chemotherapy, 39.9% of patients in the highest dose per kilogram of LBM group experienced DLT, with 25% developing neuropathy, whereas only 8.3% of patients in the lowest dose group experienced DLT, and no cases of neuropathy were observed (25). Similarly, Huemer et al. demonstrated in patients with gastric and gastroesophageal junction cancer receiving perioperative chemotherapy with the FLOT regimen (docetaxel, oxaliplatin, fluorouracil, and leucovorin) that those with baseline sarcopenia had higher drug exposure per unit LBM and a significantly increased incidence of DLT, particularly neurotoxicity and myelosuppression (26). Ongoing muscle loss during treatment was associated with poorer disease-free survival.

A population pharmacokinetic study of nab-PTX conducted by Chen et al. indicated that the clearance of nab-PTX is primarily dependent on hepatic metabolism and albumin-mediated transport (27). The clearance rate was not directly associated with muscle mass, and variations in the volume of distribution were not directly reflected in steady-state concentrations.

As early as 2017, Shachar et al. reported that patients with sarcopenic metastatic breast cancer had an increased risk of DLT associated with taxane-based chemotherapy (12). Findings from this study provide a mechanistic pharmacological explanation for this observation.

The increased susceptibility to CIPN in patients with sarcopenia may be attributed to a relative increase in dose burden per unit of LBM, resulting from a reduced volume of distribution. Further analysis of chemotherapy dose intensity per unit of LBM demonstrated that the nab-PTX dose intensity in the sarcopenia group was 14.03 mg/kg LBM, which was significantly higher than the 10.20 mg/kg LBM observed in the non-sarcopenia group (*p* < 0.001). This difference was further accentuated in the CIPN-H subgroup (14.2 vs. 10.9 mg/kg LBM, *p* = 0.034). These findings are consistent with results reported in previous studies. Guo et al. enrolled 32 patients receiving nab-PTX chemotherapy and categorized them into three groups based on nab-PTX dose intensity per unit of LBM: low LBM group (15.18 mg/kg LBM), medium LBM group (12.82 mg/kg LBM), and high LBM group (11.14 mg/kg LBM) (13).

The corresponding incidences of CIPN-H in these groups were 61.5%, 20.0%, and 11.1%, respectively. Youn et al. included 152 patients receiving first-line gemcitabine combined with nab-PTX (28). The study demonstrated that, among patients receiving higher doses of nab-PTX per kilogram of LBM (range: 0.98–8.76 mg/kg LBM), a 28-day chemotherapy cycle with a dose intensity of 125 mg/m² was used, and the threshold for the development of peripheral neuropathy was identified as 5.83 mg/kg LBM. In this study, a planned nab-PTX dose of approximately 11 mg/kg LBM was observed among patients with CIPN-H in the non-sarcopenia group. However, because dose modifications, treatment delays, RDI, and actual cumulative delivered dose were not systematically captured, this value should be interpreted as an exploratory signal rather than a validated clinical threshold. Future studies should evaluate actual delivered dose intensity per kg LBM and validate whether this cutoff can guide individualized nab-PTX dosing.

In this study, LBM demonstrated a statistically significant progressive decrease over successive chemotherapy cycles, which was accompanied by a corresponding increase in chemotherapy dose intensity per unit of LBM. Although the magnitude of this increase was not statistically significant, once this value reached or exceeded the threshold for nab-PTX-induced CIPN, it was associated with a clinically significant increase in both the incidence and severity of CIPN.

A study conducted by Aichi et al. in 2025, which included 98 patients with endometrial cancer, further supported the association between chemotherapy dose intensity per unit of LBM and CIPN (29). In that study, conventional PTX chemotherapy was administered, and PTX dose (mg/kg LBM) was significantly associated with the incidence of CIPN (OR = 2.58, 95% CI: 1.08–6.19). The incidence of CIPN was significantly higher in patients who reached or exceeded the threshold of 8.12 mg/kg compared with those below this threshold (52.0% vs. 30.1%, *p* = 0.049).

A 2025 sub-analysis of the MiroCIP study conducted by Misawa et al. followed 131 patients receiving taxane-based chemotherapy over a 12-month period and identified the temporal pattern of CIPN occurrence (30). The grade ≥ 2 CIPN peaked at approximately three months after initiation of taxane chemotherapy and subsequently reached a plateau. This peak timing is consistent with the three-cycle assessment window (approximately 9 weeks) used in this study. These findings have important clinical implications, indicating that follow-up schedules based on patient-reported outcomes may be aligned with the temporal pattern of CIPN occurrence to improve clinical management.

The kinetic–pharmacodynamic model developed by Mehrotra et al., based on data from the CALGB 40502 trial, further supports this trajectory from a quantitative pharmacological perspective (31). The model demonstrated that the longitudinal accumulation of nab-PTX-induced CIPN follows an indirect response model, in which the effect of drug concentration on neural injury exhibits a linear relationship, and a predictable quantitative association exists between early cumulative dose and neuropathy scores at three months. From these findings, dose titration guided by early indicators of CIPN (cycles 1–2) has been proposed as a strategy to reduce the risk of late-onset severe neuropathy. Overall, the accelerated accumulation pattern observed in the sarcopenia subgroup in this study provides additional temporal evidence supporting the association between body composition and CIPN and suggests that dose adjustment strategies based on LBM may be considered in patients with sarcopenia.

The multivariable analysis in this study demonstrated, in a nab-PTX cohort, a clear dose–response relationship between baseline FFMI and the risk of severe CIPN. When compared with the low FFMI group, the high FFMI group exhibited a 63.6% reduction in relative risk, a 36.8% reduction in absolute risk, and a NNT of 2.7. The area under the ROC curve (AUC = 0.682) indicates moderate discriminative ability, supporting the use of FFMI as a clinical risk stratification tool. The protective effect of FFMI may be explained by several mechanisms. First, with respect to drug distribution volume, individuals with higher FFMI have lower drug exposure per unit of LBM, consistent with the dose per unit LBM findings reported above (12, 32). Second, regarding metabolic buffering capacity, skeletal muscle serves as an important modulator of systemic inflammation and oxidative stress (33, 34).

Experimental studies have indicated that skeletal muscle secretes antioxidant proteins such as superoxide dismutase 3, and axonal degeneration is highly sensitive to oxidative damage. Third, in terms of functional reserve, FFMI is often positively associated with performance status, and individuals with higher FFMI may demonstrate improved tolerance to chemotherapy and better treatment adherence (35). Assessment of FFMI using BIA has practical clinical applicability. The identification of patients with FFMI <17.55 kg/m² as a high-risk group for CIPN supports the implementation of preventive strategies. A study conducted by Saito et al. in 2025 in patients with unresectable pancreatic cancer demonstrated that early prophylactic dose optimization of nab-PTX (within 4 weeks) in patients with low SMM significantly delayed and reduced the incidence of grade ≥ 2 CIPN, further supporting these findings (36).Although lower FFMI was associated with a higher risk of CIPN-H, the discriminatory performance of FFMI was only moderate, with an AUC of 0.682. Therefore, the proposed FFMI cutoff and risk categories should be interpreted cautiously as exploratory findings and require validation in larger independent cohorts before being used for clinical risk stratification.

Multivariable Cox regression analysis demonstrated that the risk of death in patients with severe CIPN was 3.46 times higher than that in patients with mild-to-moderate CIPN (95% CI: 1.63–7.35, *p* = 0.001), with a reduction in median OS of 11 months (13 vs. 24 months, log-rank *p* = 0.022). This association was independent of tumor type and the use of platinum-based combination chemotherapy. These findings are consistent with a systematic review by Seretny et al., which included 38 clinical trials and 4,089 patients and demonstrated that CIPN not only adversely affects quality of life but may compromise tumor control through chemotherapy dose reductions, treatment delays, or discontinuation, ultimately resulting in poorer survival outcomes (37).

Dorsey et al. further reported that moderate-to-severe CIPN represents one of the most common causes of premature discontinuation of chemotherapy, and in patients with advanced malignancies, such treatment interruptions are directly associated with reduced survival (38). Although adjustments were made for tumor type and chemotherapy regimen in the multivariable analysis, the causal relationship between CIPN-H and overall survival should be interpreted with caution. CIPN-H may influence survival indirectly through treatment modifications such as dose reductions or interruptions, or it may represent a concurrent manifestation of higher disease burden. Further studies are required to clarify the causal pathway, including the application of mediation analysis.

Limitations and directions for clinical translation: Several limitations of this study should be acknowledged. First, the single-center design and small sample size (n = 56) may introduce selection bias. Second, although CIPN-H was independently associated with poorer OS, the observational design of this study precludes causal inference. CIPN-H may be a surrogate marker of disease severity, treatment intensity, tumor burden, reduced treatment tolerance, or treatment modification. In addition, detailed data on relative dose intensity, chemotherapy dose reduction, treatment delay, and reasons for treatment discontinuation were not systematically collected. Therefore, residual confounding could not be fully excluded, and the survival findings should be interpreted as exploratory and hypothesis-generating. CIPN assessment was conducted using a patient-reported questionnaire. Although this approach is clinically practical, it lacks objective electrophysiological validation, such as nerve conduction studies, and may be subject to reporting bias. Fourth, the RDI of chemotherapy and the reasons for dose modifications were not recorded, precluding differentiation between dose reductions attributable to CIPN and those due to other causes. Fifth, the follow-up duration was limited to three chemotherapy cycles, and therefore the long-term progression and recovery of CIPN remain uncertain.

## 5 Conclusion

In patients receiving nab-PTX chemotherapy, progressive skeletal muscle loss during treatment was closely associated with an increased incidence of CIPN-H. Higher baseline FFMI was identified as an important protective factor against severe CIPN, whereas CIPN-H was identified as an independent indicator of poor prognosis. These findings support several clinical implications. Prior to the initiation of nab-PTX treatment, body composition should be assessed using L3SMI derived from routine abdominal CT imaging or by BIA. Patients with FFMI < 16.0 kg/m² or L3SMI below the Asian consensus cutoff should be considered at high risk for CIPN.

Muscle preservation strategies, including nutritional support and exercise interventions, should be incorporated as integral components of supportive care throughout the chemotherapy course. Such approaches may contribute to the prevention of CIPN and the improvement of quality of life and survival outcomes in patients.

## 6 Future perspectives

The LEANOX trial published in 2025 demonstrated that, in patients with stage III colon cancer receiving adjuvant chemotherapy, an individualized oxaliplatin dosing strategy based on LBM in those with sarcopenia significantly reduced the incidence of grade ≥ 2 oxaliplatin-induced peripheral neuropathy (OIPN), delayed its onset, and improved grade ≥ 2 OIPN-free survival and quality of life, without compromising relapse-free survival or OS (11). In future, clinical studies assessing individualized nab-PTX dosing strategies based on LBM should be further pursued, with the aim of reducing the incidence and severity of CIPN while maintaining relapse-free survival and OS.

## Data Availability

The original contributions presented in the study are included in the article/**Supplementary Material**. Further inquiries can be directed to the corresponding author.

## Glossary

ALT: Alanine aminotransferase
ARR: Absolute risk reduction
AST: Aspartate aminotransferase
AUC: Area under the curve
AWGS: Asian Working Group for Sarcopenia
BIA: Bioelectrical impedance analysis
BMI: Body mass index
BSA: Body surface area
CAPOX-BEV: Capecitabine, oxaliplatin, and bevacizumab
CI: Confidence interval
CIPN: Chemotherapy-induced peripheral neuropathy
CIPN-H: High-grade chemotherapy-induced peripheral neuropathy (≥ grade C)
CIPN-L: Low-grade chemotherapy-induced peripheral neuropathy (< grade C)
CT: Computed tomography
DLT: Dose-limiting toxicity
FFMI: Fat-free mass index
FLOT: Docetaxel, oxaliplatin, fluorouracil, leucovorin
HR: Hazard ratio
KPD: Kinetic-pharmacodynamic
KPS: Karnofsky Performance Status
L3SMI: L3 skeletal muscle index
LBM: Lean body mass
nab-PTX: Albumin-bound paclitaxel
NNT: Number needed to treat
NRS2002: Nutritional Risk Screening 2002
OIPN: Oxaliplatin-induced peripheral neuropathy
OR: Odds ratio
OS: Overall survival
PFS: Progression-free survival
PG-SGA: Patient-Generated Subjective Global Assessment
PK: Pharmacokinetics
PNQ: Patient Neurotoxicity Questionnaire
PTX: Paclitaxel
RDI: Relative dose intensity
ROC: Receiver operating characteristic
L3SMA: Skeletal muscle area at L3
SMM: Skeletal muscle mass
SOD3: Superoxide dismutase 3
TPS: Treatment Planning System
ULN: Upper limit of normal
