# Supplementary material for: Prospective evaluation of dynamic changes in L3 skeletal muscle index and chemotherapy-induced peripheral neuropathy during albumin-bound paclitaxel chemotherapy
Source: Front Oncol. 2026 Jul 27;16:1844651. doi: 10.3389/fonc.2026.1844651 (PMC13453689; doi:10.3389/fonc.2026.1844651)
Supplement: Supplementary file 1 [file Table1.docx]

Supplementary Table 1: sensibility analysis Between G1 and G2

| Variable | G1 | G2 | G1-IQR | G2-IQR | Primary_paired_t_test_P | Sensitivity_P |
| --- | --- | --- | --- | --- | --- | --- |
| NRS2002 | 2.45 ± 1.70 | 2.55 ± 1.93 | 2.00 (1.00–3.00) | 2.00 (1.00–3.00) | 0.585 | 0.511 |
| PG-SGA | 7.59 ± 5.14 | 8.27 ± 5.41 | 7.00(3.00–10.00) | 7.00 (4.00–11.25) | 0.25 | 0.296 |
| BMI | 22.68 ± 3.43 | 22.70 ± 3.51 | 22.80(20.51–24.79) | 22.72 (20.29–25.13) | 0.934 | 0.899 |
| L3SMA | 93.51± 26.89 | 91.06± 25.83 | 89.65(74.47–109.65) | 87.57 (72.11–108.25) | 0.048 | 0.115 |
| L3SMI | 32.77 ± 8.72 | 31.93 ± 8.46 | 31.77 (26.21–38.18) | 30.83 (25.73–38.62) | 0.049 | 0.113 |
| LBM | 34.11 ± 8.07 | 33.38 ± 7.75 | 32.95 (28.40–38.96) | 32.33 (27.69–38.53) | 0.047 | 0.115 |
| FFMI | 17.07 ± 2.04 | 16.63 ± 2.23 | 17.05 (15.58–18.39) | 16.95 (15.15–18.12) | 0.025 | 0.079 |
| BSA | 1.73 ± 0.15 | 1.73 ± 0.16 | 1.75 (1.62–1.84) | 1.73 (1.64–1.83) | 0.841 | 0.989 |
| CIPN-H | 6/56 (10.71%) | 22/56 (39.29%) | NA | NA | NA | <0.001 |

Note: the baseline chemotherapy group (Group G1) ; the post-chemotherapy group (Group G2)

Supplementary Table 2: Univariate Cox Regression Analysis of Overall Survival

| Variable | HR | 95% CI Lower | 95% CI Upper | P-value |
| --- | --- | --- | --- | --- |
| Gender (Gender Female) | 0.966 | 0.438 | 2.13 | 0.932 |
| Age (years) | 0.968 | 0.922 | 1.017 | 0.2 |
| NRS2002_baseline | 1.064 | 0.925 | 1.224 | 0.387 |
| PG-SGA Scale | 1 | 0.941 | 1.062 | 0.991 |
| L3SMA_baseline | 0.996 | 0.983 | 1.009 | 0.523 |
| L3SMI_baseline | 0.982 | 0.944 | 1.022 | 0.382 |
| Height (cm) | 1.013 | 0.969 | 1.059 | 0.562 |
| Weight baseline (kg) | 1.001 | 0.971 | 1.032 | 0.931 |
| FFMI_baseline | 0.928 | 0.782 | 1.101 | 0.389 |
| LBM_baseline | 0.986 | 0.945 | 1.029 | 0.523 |
| BSA_baseline | 1.422 | 0.174 | 11.628 | 0.743 |
| Body Mass Index（kg/m2）baseline | 0.991 | 0.896 | 1.095 | 0.855 |
| CIPN_H | 2.099 | 1.104 | 3.99 | 0.024 |
| medication_duration (medication_durationsecond line) | 1.415 | 0.604 | 3.314 | 0.424 |
| medication_route (medication_routeone day) | 0.898 | 0.465 | 1.736 | 0.75 |
| sarcopenia_baseline (sarcopenia_baselineNon_Sarcopenia) | 1.076 | 0.567 | 2.043 | 0.823 |
| Cancer Type | 3.719 | 1.043 | 13.258 | 0.043 |
| Treatment Regimen(platinum_combinedYes) | 0.545 | 0.267 | 1.11 | 0.094 |
| Cancer Type (Lung cancer) | 0.619 | 0.314 | 1.222 | 0.167 |
